# Supplementary material for: Are carbon emissions trading and green financial instruments synergistic? -Comprehensive quantitative research based on content analysis
Source: PLoS One. 2024 Mar 7;19(3):e0298601. doi: 10.1371/journal.pone.0298601 (PMC10919601; doi:10.1371/journal.pone.0298601)
Supplement: S1 Data — (ZIP) [file pone.0298601.s001.zip › Data/地级市Z_F工作报告.zip!/╕╩╦α/╛╞╚¬╩╨/└┤╫╘╣1⁄2╓┌║┼í╛┬φ┐╦╩2╛▌═°í┐_2019.docx]

各位代表：

现在，我代表市人民政府向大会报告工作，请予审议，并请各位政协委员和列席人员提出意见。

2018年工作回顾

过去的一年，面对外部环境复杂多变和经济下行压力加大的严峻形势,在省委、省政府的正确领导和市委的坚强领导下，我们认真贯彻习近平新时代中国特色社会主义思想和党的十九大精神，全面落实习近平总书记视察甘肃重要讲话和“八个着力”重要指示精神，坚持稳中求进工作总基调，认真落实市委“八个走前列”工作部署，攻坚克难，担当实干，统筹稳增长、促改革、调结构、惠民生、防风险各项工作，经济运行持续向好，高质量发展迈出坚实步伐。初步统计，全年实现生产总值596.9亿元，增长4.6%；固定资产投资增长7.2%；社会消费品零售总额188.6亿元，增长7.3%；一般公共预算收入36.5亿元，增长5.9%；城乡居民人均可支配收入分别达到35239元和17104元，均增长8.5%。除地区生产总值、社会消费品零售总额增速低于目标0.4和0.7个百分点外，其他预期指标超额完成市四届人大二次会议确定的目标任务。

——政策集成落地更加有效。认真落实中央和省委、省政府稳增长、补短板、强弱项等政策措施，出台了加快推进“八个走前列”实施意见、工业企业复产达标升规行动计划、全域旅游强市建设、融入“一带一路”发展外向型经济、加强精准招商促进经济稳定增长等一系列政策文件，推出了重大产业分组推进、重点企业“一企一策”帮扶等一系列工作举措，落实了精准脱贫攻坚、生态环保问题整改、绿色产业培育、债务风险防控、营商环境改善等一系列重点工作，形成了上下衔接、协同配套、健全完善的政策体系和工作推进机制，最优政策组合和整体效果充分显现，为经济稳定增长提供了有力支撑。

——经济发展基础更加坚实。坚持把扩大有效投资和培育工业企业作为推进高质量发展的两个重要抓手，落实市级领导包抓重大项目、带头外出招商制度，一批基础设施和产业项目开工建设，投资保持较快增长。敦格铁路酒泉段、马鬃山口岸公路建成通车，敦当高速、柳敦高速加快推进，敦煌城市水源置换、肃州洪水河水库等项目开工。肃州碧桂园商住综合体等一批招商项目落地建设，实际到位资金313.8亿元。加强工业经济运行调度，对128户企业实行包抓帮扶，落实酒泉核技术产业园、玉门油田公司、三新硅业“一企一策”支持措施，瓜州常乐电厂土建主体完工，酒泉经开区主要指标全面回升，玉门经开区获批省级高新技术开发区，金塔工业集中区升格为省级开发区，新增高新技术企业21户，东方铝业、祁连山药业等3户企业新认定为省级战略性新兴产业骨干企业，总数达到12户。新增规模以上工业企业21户，规上工业增加值增长3.9%，主营业务收入增长16.5%。

——绿色产业导向更加鲜明。制定实施构建十大生态产业体系推动经济高质量发展政策措施，推进86个总投资约1450亿元的重大带动性项目建设。清洁能源产业调整优化，国内首个光热发电示范项目首航节能敦煌100兆瓦熔盐塔式光热电站并网发电，落实直购电交易6.6亿千瓦时，风电、光电上网率分别达到76.8%和88.3%。文旅产业快速发展，敦煌游客接待中心建成投用，金塔胡杨文化旅游节、瓜州玄奘之路戈壁挑战赛等活动带动效应增强，实现旅游接待人数3235万人次、旅游收入316.3亿元、文化产业增加值20亿元，分别增长22.1%、29.1%和12.5%。装备制造、清洁生产、节能环保产业稳步发展，玉门润泽环保固废处理、金塔欧力特石油钻具等项目加快建设，瓜州、金塔清洁供暖扩容项目建成。军民融合、数据信息、中医中药产业加速培育，酒泉核技术产业园完成年度建设任务，甘肃佛慈制药综合开发项目落地实施。

——生态环保治理更加有力。全面推进各类生态环保问题整改，中央环保督察、省级环保督察、自然保护区排查问题整改率分别达到100%、86.7%和90.2%。制定祁连山国家公园体制试点实施方案，范围勘界落界、自然资源本底调查稳步推进。深入实施蓝天、碧水、净土三大保卫战，淘汰燃煤锅炉228台，改造土炕和燃煤小火炉2.5万户，主要污染物减排目标全面完成。肃州、金塔、玉门、敦煌再生水利用项目开工建设，11个工业集中区和38个乡镇污水处理设施建成，“河长制”“湖长制”全面落实，地表水、地下水监测达标率分别为100%和83.3%。国土绿化行动持续推进，完成人工造林、封滩育林36.6万亩，我市被列为全国防沙治沙综合示范区。

——乡村振兴起步更加稳健。巩固提升脱贫攻坚成果，落实“一户一策”措施，整合投入扶贫资金2.3亿元，支持发展特色扶贫产业，移民人均可支配收入增长10.3%，减少贫困人口2604人，瓜州县脱贫摘帽。出台戈壁生态农业发展规划及政策扶持办法，新建戈壁生态农业3.8万亩，中国-以色列（酒泉）绿色生态产业园获省政府批准建设，戈壁生态农业经验做法受到国务院和省政府通报表扬。发展优势特色产业221万亩，肉羊养殖达到750万只，“戈壁雪润”区域公用商标注册启用，新认证“三品一标”农产品36个，发展农业新型经营主体258家，一产增加值增长7.3%。狠抓“三沿一部”环境整治和全域无垃圾专项治理，新改建农村公路980公里，改造农村住房5268户、家庭卫生户厕3.1万户。

——改革开放成效更加明显。供给侧结构性改革深入推进，退出煤炭产能66万吨，商品房去化周期保持在合理范围。政府债务全面核查备案，债务规模控制在限额以内。市级行政审批事项削减54.4%，酒泉市行政服务中心扩建搬迁，玉门市“一处跑、跑一次”改革经验在全省推广。实施“多证合一”商事制度改革，新增各类市场主体1.67万户，电商交易额实现86亿元，增长25%。农村“三变”改革试点稳步推进，酒泉经开区管理体制理顺，经投集团重组改革加快，玉门油田和农垦企业“三供一业”正式移交。浙商银行、省产权交易所在酒泉设立分支机构，地方金融业态进一步规范，金融风险总体可控。第三届敦煌文博会成功举办，开展论坛、展览等活动30多场次，荣获“十大政府主导型展览会”大奖。企业“走出去”步伐加快，恒亚水泥、神舟农业科技等企业在“一带一路”沿线国家设立生产基地。

——城市功能配套更加完善。酒泉中心城区控制性详规和“城市双修”等专项规划批复实施。全市新建改造市政道路47.8公里，新增公共绿地150万平方米，实施棚户区改造8329户。酒泉城区改造提升三年行动全面启动，路网畅通、立面改造、文化挖掘有序推进，酒银大桥、南滨河路、美溪公园一期建成。金塔新城区建设加快，敦煌飞天公园、肃北党河东岸管网改造、阿克塞第二热源厂等市政工程建成。市级城市管理执法机构组建，“文明出行”专项行动取得实效，城市网格化、精细化管理水平提高。玉门、敦煌获得全国文明城市提名资格，瓜州、肃北创建国家园林县城通过省级验收。

——民生事业保障更加充分。财政投入民生保障资金101.5亿元，占一般公共预算支出的74%。新增天然气用户3万户、新改建标准化公厕157座等为民实事完成。教育惠民政策全面落实，新建改造校舍14.5万平方米，酒泉七中建成招生，新增6所省级示范性幼儿园，累计达到11所。文化服务体系不断健全，市体育馆、文化中心剧场和铁人王进喜纪念馆投入使用，七个县市区融媒体中心挂牌。加快补齐医疗短板，玉门市第一医院建成投用，市妇幼保健服务中心、市二院综合大楼主体完工，“先看病后付费”结算全面推开。就业和社会保障水平提高，城镇新增就业2.2万人，输转劳动力18.6万人，城乡低保标准分别提高8%和22.6%，特困供养标准提高7.6%，城乡居民养老保险参保率达98.4%，肃州、玉门成为首批全省医养结合示范先行县市。“扫黑除恶”取得阶段性成果，刑事发案同比下降6.3%。安全生产形势平稳，食品药品安全治理成效明显。民族宗教政策全面落实，我市创建为全省民族团结进步示范市。防震减灾、消防安全、双拥共建、人防建设、气象服务继续加强，妇女儿童、残疾人和老年人权益有效保障，档案、史志、侨务等事业取得新的成绩。

各位代表！

过去的一年，我们坚持以党的建设统领各项工作，牢固树立“四个意识”，坚定“四个自信”，践行“两个维护”，全面彻底肃清王三运、马光明流毒和影响。自觉接受人大法律监督和工作监督、政协民主监督及社会各界监督，人大代表建议和政协委员提案全部答复办结。加强政府立法工作，首部地方性法规《酒泉市饮用水水源地保护条例》颁布实施。加强审计监督，查处纠正违纪违规和管理不规范资金8.5亿元。深入开展“作风建设年”“放管服改革突破年”活动，取得了高质量发展的新成效。这些成绩来之不易，得益于省委、省政府和市委的坚强领导，得益于市人大、市政协和社会各界的监督支持，得益于全市干部群众的共同努力。在此，我代表市人民政府，向全市各族人民，向人大代表、政协委员、各民主党派、工商联、人民团体致以崇高的敬意！向驻酒人民解放军、武警官兵、人民警察、中央省属驻酒单位，以及所有关心支持酒泉发展的社会各界人士表示衷心的感谢！

综合分析，当前我市经济社会发展中还存在一些突出矛盾和问题。一是发展制约因素增多。土地、环保、能耗等要素趋紧，招商引资难度加大，本土企业发展困难，民间投资意愿下降，经济持续增长支撑乏力。二是新旧动能转换接续不足。工业企业停减产较多，石油、电力行业增速回落，企业复产升规难度较大；农业龙头企业带动力不强，现代农业体系不完善、整体效益不高。三是生态环境治理任务繁重。全市自然保护区个数占全省四分之一、面积占全市的四分之一，环保问题整改和生态修复难度大，环保设施建设滞后，打赢污染防治攻坚战任务艰巨。四是社会事业发展不够充分。民生领域欠账和遗留问题较多，教育、医疗、养老、社会保障等方面，与人民群众的期盼还有差距。五是政府系统作风转变还不到位。一些干部服务意识不强，担当精神不够，工作落实不力,庸懒散慢现象依然存在，行政服务效能还有待提高。对这些问题，我们不回避、不遮掩、不懈怠，将采取有力措施，认真解决。

2019年重点工作

今年，是新中国成立70周年，是决胜全面建成小康社会的关键之年。我们要准确把握重要战略机遇期、动能转换窗口期、重大任务攻坚期，过好高质量发展的战略定力关、破解难题的攻坚能力关、解放思想的干事魄力关，坚定信心，迎难而上，全力以赴做好改革发展稳定各项工作。按照中央和省委、市委经济工作会议的部署，政府工作的总体要求是：以习近平新时代中国特色社会主义思想为指导，全面贯彻党的十九大和十九届二中、三中全会精神，统筹推进“五位一体”总体布局，协调推进“四个全面”战略布局，深入落实习近平总书记视察甘肃重要讲话和“八个着力”重要指示精神，坚持稳中求进工作总基调，牢固树立新发展理念，以高质量发展为方向，以供给侧结构性改革为主线，按照“八个走前列”、建设省域副中心城市的目标要求，进一步解放思想、改革创新，加快建设现代产业体系，深入实施乡村振兴战略，继续打好“三大攻坚战”，持续做大经济总量，提升经济实力，提高发展质量，统筹推进稳增长、促改革、调结构、惠民生、防风险各项工作，为全面建成小康社会收官打下决定性基础，以优异成绩庆祝中华人民共和国成立70周年。

预期目标是：生产总值增长6.2%左右，固定资产投资增长8%，社会消费品零售总额增长8%，一般公共预算收入同口径增长6%，城镇居民人均可支配收入增长7%，农村居民人均可支配收入增长8%，居民消费价格指数涨幅控制在3%以内，单位生产总值能耗和主要污染物排放控制在省定目标以内。实现上述目标，我们将重点抓好八个方面的工作：

（一）加快构建现代产业体系，培育高质量发展新动能。坚持绿色引领，聚焦生态产业，加快建设主导产业清晰、比较优势明显、集聚效应突出的现代产业体系。

加快发展十大生态产业。坚持把绿色生态产业作为加快新旧动能转换的重要抓手，推动重大带动性项目建设，力促绿色生态产业实现“大发力”。做强做优循环农业、清洁能源、文化旅游、装备制造4个优势类产业，力争增速高于生产总值，使其成为推动高质量发展的支柱产业。做大做好通道物流、军民融合、清洁生产、节能环保4个基础条件较好产业，加大政策引导和扶持力度，加快培育成为新的经济增长点。做实做足数据信息、中医中药2个成长型产业，谋划建设一批引领性项目，着力打造地方经济发展新亮点。

推进新能源健康发展。开工建设风电二期二批部分项目，加快常乐电厂建设进度，建成玉门鑫能、敦煌大成等光热发电示范项目和中能智慧化学储能电站，争取玉门昌马120万千瓦抽水蓄能电站获批建设。提升电能外送和就地消纳能力，确保河西750千伏第二通道加强工程和肃州区新能源微电网示范项目建成投运。落实清洁取暖优惠电价政策，加快推进清洁能源供暖改造。

支持优势产业转型升级。加大矿产冶炼、石油化工、建筑建材等产业绿色化、智能化、清洁化改造力度，建设玉门、金塔、瓜州现代煤化工示范工程，实施三新硅业、万象建材、恒亚水泥等用能大户节能改造，推进玉门油田10万吨高端低凝液压油、80万吨重油催化裂化装置节能改造等项目建设。整合重组金塔、瓜州、敦煌石材产业，加快石材产业转型和加工链延伸。促进恒润5万吨保温材料、天鸿2万吨高性能玻璃棉、玉门华事达等建材企业稳产增效，提高资源综合利用水平。全面落实企业复产达标升规和工业创新发展激励政策，力争新培育规上工业企业30户。

持续培育战略新兴产业。扎实开展先进制造业发展专项行动计划，建成凯盛大明聚光材料、大禹农业精量灌溉装备智能制造等项目，推进中材科技工装电技术改造和东方铝业装备制造生产线建设。全力保障酒泉核技术产业园建设，加快罗普特军民融合安全产业园项目进度，争取百立通用机场立项实施。建成酒泉云计算（大数据）中心一期项目主体工程，加快政务云、企业云平台建设。加大中医中药产业培育力度，支持枸杞、甘草、苁蓉等地产药材精深加工，扶持祁连药业、大得利药业、甘帝药业等企业开发新品，加快佛慈药业综合开发项目建设。

（二）扎实推进乡村振兴战略，形成高质量发展新优势。坚持把乡村振兴战略作为“三农”工作的总抓手，聚焦产业、人才、文化、生态、组织“五个振兴”，全面落实乡村振兴战略规划，力促农业增效、农民增收、农村进步。

振兴特色优势产业。把产业振兴作为推进乡村振兴的首要任务，按照“扩规模、引龙头、建体系、育品牌、增效益”的思路，提升壮大高效蔬菜、现代制种、名优林果、特色中药材、草食畜五大优势产业，发展高效蔬菜50万亩、现代制种50万亩、优质林果40万亩、特色中药材45万亩。推进牧草基地和智慧牧场建设，新增肉羊出栏50万只，建设佛慈产业园万头驴养殖基地，创建国家级蔬菜花卉制种基地和绿色有机牛羊肉生产示范县。

发展戈壁生态农业。以发展“高端、绿色、有机”农产品为方向，支持山东水发、海升集团、敦煌种业、大禹节水等龙头企业加快建设戈壁生态农业产业园区，积极落实特色产业工程贷款、担保贴息、农业保险等政策措施，新建戈壁生态农业4万亩，重点在肃州、玉门、金塔集中布局，连片建设一批千亩以上标准化产业园区。全面实施中以(酒泉)绿色生态产业园建设,落实省级开发区优惠政策，积极引进以色列和其他国家成套生产技术与管理经验，加快推进高标准连栋示范温室和连片日光温室建设，着力打造科技含量高、创新能力强的绿色生态产业园和国际合作示范基地。

构建产业化经营体系。坚持质量兴农、绿色惠农、品牌强农，推进种植养殖、市场营销、质量监管、品牌支撑、科技服务为一体的全产业链开发。培育壮大产业化龙头企业，实施农民专业合作社“能力提升工程”，扶持发展农产品精深加工，新增冷链保鲜仓储能力15万吨。支持优势企业在国内大中城市和“一带一路”沿线国家设立销售窗口，办好特色农产品展销会、第二届酒泉种子博览会等促销节会。做强“戈壁雪润”区域公共品牌，健全农产品质量监管追溯体系，新认证“三品一标”农产品20个以上，发展“三品一标”优质农产品120万亩以上。

推进城乡融合发展。深入挖掘农村生态涵养、休闲观光、文化体验、健康养老等功能，实施休闲农业和乡村旅游精品工程，打造阳关旅游休闲小镇、玉酒泉文化小镇等10个特色小镇，加快泉湖镇永久村、金塔镇红光村等10个田园综合体建设，推进农村一二三产业融合发展。实施“互联网+现代农业”行动，完善农村电商服务体系，乡村电商站点覆盖率分别达到100%和80%。坚决彻底完成“大棚房”清理整治，严禁农地非农化问题发生。

创新农村体制机制。健全农村产权交易平台，保障农村土地、林地等权属有序合法流转。推广农村“三变”改革试点经验，鼓励新型经营主体参与农村“三变”改革，为乡村振兴注入新活力。推进农村房屋不动产登记，探索宅基地“三权”分置改革，盘活用好闲置农房和宅基地，促进资源要素合理流动和优化配置。创新生产经营机制、社会服务体系和产业组织模式，把小农户引入现代农业发展大格局。引导金融机构优化乡村网点布局，全面推开“两权”抵押贷款业务。

（三）提升壮大现代服务业，强化高质量发展新支撑。抢抓“一带一路”机遇，聚焦文化、枢纽、技术、信息、生态五个制高点，激发消费潜力，培育新兴业态，推进现代服务业加快发展。

聚力大敦煌文化旅游经济圈。坚持世界级定位、一体化打造，编制完成大敦煌文化旅游经济圈发展规划。实施道路设施联通工程，开工建设敦煌景观大道旅游廊道、双塔至石包城、肃北至沙枣园等旅游公路，改造鸣沙山·月牙泉景区东西出入口连接线。加快建设莫高窟游客服务中心二期、悬泉置世界文化遗产景区、榆林窟－锁阳城文化景区、肃北蒙古大营、阿克塞沙漠公园等项目，提升敦煌、瓜州、肃北、阿克塞西线景区品质品位，培育辐射河西及青海、新疆等周边地区旅游线路，形成“一核多圈”的大敦煌文化旅游发展格局。推进敦煌国家级旅游改革创新先行区、敦煌国家级文化产业示范园区建设。

推进文旅融合创新发展。深度挖掘敦煌文化、丝路文化、航天文化等特色文化内涵，高品质开发酒泉航天太空城、瓜州国际雕塑艺术公园等项目，加快肃州湿地大景区、玉门魔山地质公园建设，启动金塔沙漠胡杨林5A级景区创建工作。举办酒泉边塞文化旅游节，办好金塔胡杨文化旅游节、国际戈壁超级马拉松、玄奘之路戈壁挑战赛等节会，旅游人数和旅游收入分别增长15%和20%以上。扶持文化创意、会展演艺、数字动漫等业态发展壮大，增强《丝路花雨》《又见敦煌》等经典剧目吸引力、传播力，做大文化市场主体，文化产业增加值增长20%以上。

全力推动服务业提档升级。以产业转型和需求升级为导向，加快生产性服务业专业化发展，实施兰铁酒泉物流园区、酒泉高铁站商贸中心等项目，改造提升春光市场，建成上亿西部商贸城。加快商贸流通、餐饮娱乐等传统产业提档升级，推动建设便利店、社区菜店等社区商业和农村集镇商贸中心。落实“互联网+流通”行动计划，加大网货品牌站点、电商龙头企业扶持力度，电商交易额增长25%以上。开展家具家电惠民行、春夏汽车展等促销活动，引导农村居民增加通讯、汽车等领域消费，支持自驾游、旅游住宿等大众消费，加快发展现代金融、信息科技、医养保健等现代服务业，第三产业增加值增长8%以上。

（四）继续打好三大攻坚战，落实高质量发展新要求。坚持底线思维，扛起政治责任，抓重点、补短板、强弱项，为决胜全面建成小康社会提供坚强保障。

强力推进生态环保问题整改。从严从实推进中央和省级环保督察、“绿盾”行动等反馈问题整改，完成探采矿权退出和生态环境修复年度任务，制定完善验收标准和办法，加快推进问题整改销号，确保各类生态环保问题限期整改到位。做好生态红线勘界定标工作，严格生态功能区边界管控，积极推进祁连山国家公园体制试点相关工作。持续开展市级环保督察，巩固提升整体治理成效，加强生态环境保护长效机制建设，坚决守住环境安全底线，切实保障人民群众的环境权益。

持续开展污染防治攻坚。坚决打好蓝天、碧水、净土保卫战，加强高耗能行业污染排放和能耗管控，确保实现能耗“双控”和污染减排目标。深入开展饮用水水源地环境保护专项行动，严厉打击固体废物及危险废物非法处置行为。加快补齐环保设施短板，继续推进酒泉城区生活垃圾分类处理试点，工业园区污水处理设施达标稳定运行，有条件的农村集镇全面建成污水处理设施。加强农村面源污染治理，抓好酒泉花城湖、金塔北海子国家湿地公园建设和湿地保护与修复。稳步推进三北防护林五期、沙化土地封禁、新一轮退耕还林等工程，争取黑河流域百万亩防护林基地项目获批实施。开展大规模国土绿化行动，完成人工造林13万亩、封滩育林15万亩。积极推进国家生态文明示范市创建，加快构建生态文明体系。

巩固提升精准脱贫成果。深入推进脱贫攻坚三年行动，聚焦“两不愁、三保障”，针对未脱贫和潜在返贫人口，抓实抓细“一户一策”帮扶措施。因地制宜发展特色优势产业，不断提升贫困村基础设施条件和公共服务水平，实施移民乡村土地改良2万亩，落实教育、健康、社保等兜底政策，管好用好扶贫资金，确保剩余贫困人口全部脱贫，确保玉门市脱贫摘帽，确保移民安置区人均可支配收入增长10%以上，坚决打赢精准脱贫攻坚战。

严控防范化解重大风险。健全完善风险防控机制，落实风险防控主体责任，提高风险防控化解能力，坚决守住不发生系统性风险的底线。加强政府债务管理，规范PPP项目实施和政府购买服务，健全政府债务动态监控机制，坚决遏制变相举债和隐性负债，推动融资平台市场化转型。防范化解金融风险，强化小额贷款、融资担保、典当行、融资租赁等非银行金融机构监管，提高预防预警预判能力，严厉打击非法集资、非法互联网金融服务、恶意逃废银行债务等行为，持续优化金融生态环境。做好意识形态、经济、科技、社会、外部环境等领域风险防控工作。

（五）加快省域副中心城市建设，打造高质量发展新高地。坚持区域协调发展，以人的城镇化为核心，全面提高城镇化发展质量和水平，努力让城乡更加美丽、更加宜居。

提升市域通达能力。抢抓基础设施领域补短板政策机遇，加快市域交通体系建设，提升对外互联互通水平。完成敦煌至格尔木铁路、敦煌铁路提速改造工程，确保柳敦高速、肃航公路酒泉东互通连接线一级公路、肃航公路至核技术产业园二级公路建成通车，实施酒泉火车站改造工程。加快敦当高速建设进度，开工建设酒额铁路肃州至酒泉卫星基地段、酒嘉绕城高速和酒泉南综合客运枢纽。做好国道312酒嘉过境段、敦煌客运枢纽、阿克塞至若羌公路前期工作。支持瓜州争创全国“四好农村路”示范县。

完善提升城市功能。推动实施一批城市修补、生态修复工程，加快建设洪水河水库，做好引哈济党可研编制。加快酒泉城区改造提升三年行动，继续实施道

路畅通、立面改造、环境美化等市政工程，建成美溪公园二期和北大河沿岸景观廊道。做靓敦煌城市名片，新建效谷路、文昌路等道路，建成一批居民小区智能充电设施。提升玉门城市品位，加快市政管网、老旧楼院和城中村改造，实施经二路、南环路建设和重点路段、水系景观绿化亮化工程。

打造精品县城集镇。加快实施瓜州、肃北、阿克塞“多规合一”规划，改造提升县城道路、水电气暖等基础设施，重点实施金塔新城区设施配套、瓜州软化水处理、肃北党河城区段综合治理、阿克塞文化风情街改造等市政工程。抓好环城林网、生态廊道、绿地水系建设，瓜州、肃北创建为国家园林县城。加快供排水管网、垃圾污水处理设施建设，确保县城生活污水提标排放，实现集镇污水处理设施和垃圾收集转运处理全覆盖。推进航天镇、花海镇、七里镇等11个全国重点镇建设，因地制宜打造特色鲜明、富有活力的农村集镇。

强化城乡环境治理。规范城市综合执法管理，着力解决占道经营、违章搭建、乱堆乱放等问题，规范商户牌匾及户外广告，拓展城市网格化管理，推行市容市貌精细化、常态化巡查。落实全域无垃圾专项治理行动和农村人居环境整治三年实施方案，学习借鉴浙江“千万工程”经验，加大“三沿一部”环境综合整治，提升重点乡镇、节点村组、高速公路服务区、农垦团场综合整治水平。全面推进农宅风貌改造、房前屋后保洁、陈年垃圾清理、生活污水处理、环境绿化美化，改造农村土坯房1.9万户。

（六）大力发展民营经济，增强高质量发展新活力。坚持“两个毫不动摇”，更好发挥民营经济对地方发展的支撑作用，让民营经济创新源泉充分涌动、创造活力充分迸发。

落实兑现各项扶持政策。认真落实国办聚焦企业关切优化营商环境26条和省委省政府支持非公经济发展19条、市委市政府支持中小微企业高质量发展70条等政策措施，细化完善市场开放、要素配置、财税支持、信贷融资、人才引进等扶持政策，精准有效支持民营经济发展。落实酒泉本土民营企业享受招商引资优惠政策，鼓励本土企业互保共建、互助促销、抱团发展。坚决破除民营企业发展障碍，加快推进市场准入负面清单管理制度，推进涉企行政事业性收费零收费，实质性降低企业税费负担和运行成本。加强舆论引导和政策解读，提高民营企业家对各项政策的知晓度。

真心帮助企业脱困解难。坚持各级领导联系非公企业制度，重视解决生产经营、资金融通、项目落地等方面的实际困难，带头构建“亲”“清”新型政商关系。深化政银企对接服务，用好金控融资担保、产业工程贷款等平台，推进股权、订单、知识产权等抵质押贷款业务，推广小微企业互助担保贷款、小额信贷保证保险等金融产品，切实解决小微企业融资难融资贵问题。拓宽民营企业融资渠道，支持有条件的中小企业通过“新三板”挂牌、产权转让等方式直接融资。依法保护民营企业物权、债权、知识产权等财产权，稳妥化解涉及民营企业的矛盾纠纷，让民营企业专心创业、放心投资、安心经营。

强力推进项目招引落地。完善精准招商措施，落实优势产业包抓方案，围绕装备制造、交通水利、现代农业、文化旅游、垃圾污水处理、节能环保等优势特色领域和基础设施短板，紧盯国内大型企业和行业龙头企业，持续开展精准招商。大力推进领导干部招商、以商招商、产业链招商，形成市县联动、部门配合、企业参与的招商引资合力。建立招商项目绿色服务通道，完善审批备案、规划选址、土地预审、环评安评、基础配套联动机制，促成落地建设一批招商项目，引进到位资金增长8%以上。

激发县域经济发展活力。扶持民营企业积极参与县域主导产业培育和名优名品开发，提高配套能力、技术含量和产品附加值，推进县域农业精品化、工业园区化、三产规模化发展。发挥民营企业在县域经济中的主力军作用，帮助有条件的企业积极争取利用生态产业发展基金，扶持壮大农产品加工、文化旅游、现代物流等特色产业，促进形成带动县域经济发展的龙头企业，非公经济占生产总值的比重提高到52%以上。

（七）深入推进改革开放创新，激发高质量发展新动力。站在改革开放40周年新的起点上，以更坚定的信心、更有力的举措，把改革开放不断推向深入，培育区域竞争新优势，为高质量发展提供强大动能。

统筹重点领域改革。全面完成市县乡机构改革，进一步理顺行政管理体制，规范事业单位运行机制。整合组建市场监管、生态环保、文化市场、交通运输、农业领域综合执法队伍，切实解决多头执法、多层执法和重复执法等突出问题。深化经济体制改革，落实好国家更大规模的减税降费政策，继续推进增量配电改革，扩大直购电交易规模。加快国有企业改革，全面完成市经投集团组建。深化“放管服”改革，落实“四办”措施，“最多跑一次”实现市县乡全覆盖，最大限度利企便民，提高办事效率。深化投融资改革，探索利用资金池、建养一体化、融资租赁、经营性租赁等模式，筹措基础设施项目建设资金。

深度融入一带一路。大力推进国际产能合作，支持万象建材、恒亚水泥在中西亚地区建设生产线。加快建设敦煌国际空港、酒嘉物流园、敦煌现代物流园，着力打造酒泉国家陆港型物流枢纽载体城市。积极融入国际陆海贸易新通道，支持龙头企业在国内外设立销售仓储专区，助力酒泉特色农产品销往珠三角、西南地区及东盟国家。鼓励我市企业从中亚进口小麦、食用油等优质产品，推进机械装备、节水材料、新能源装备等外贸出口。精心办好第四届丝绸之路（敦煌）国际文化博览会，提升内涵品质，放大品牌效应，推动会展活动常态化，打造永不落幕的文博会。

增强科技创新能力。深入实施创新驱动发展战略，依托酒泉国家农业科技园区和4个省级农业科技园区，加大科技创新、成果转化和示范推广力度。完善人才发展政策，培养用好本土人才，引进留住外部人才，夯实高质量发展人才支撑。加快创新平台建设，支持企业与高校、科研院所开展产学研合作，培育具有标志性、引领性的科技示范项目，扶持小微企业、创新团队、领军人才创办科技型企业，新增高新技术企业5家、科技型企业15家，科技进步贡献率达到60%以上。

（八）全力保障改善民生，共享高质量发展新成果。牢固树立以人民为中心的发展思想，聚焦“七有”领域，多谋利民之事，多解民生之忧，在发展中补齐民生短板，不断提高人民群众的获得感、幸福感、安全感。

提升公共服务体系。促进教育优质优先发展，着力解决学前教育短板和义务教育结构性矛盾，提高普通高中办学水平，全面消除城市中小学“大班额”，学前教育三年毛入园率达到98.5%以上，九年义务教育巩固率达到99.5%以上。加快健康酒泉建设，深化医疗卫生体制改革，加大基层全科医生培养力度，大力推进医养结合，提高医疗卫生质量。落实“基层首诊”制度，推行按病种付费分级诊疗，实现二级以上医院异地就医即时结算，建成卫生应急指挥中心和市县两级全民健康信息平台。深入实施文化惠民工程，力争开工丝绸之路文化研究中心及酒泉市图书馆，推进县级文化馆图书馆总分馆制试点建设。

健全社会保障体系。把稳就业摆在突出位置，落实企业吸纳贫困劳动力、创业担保贷款贴息、失业人员技能培训补贴等就业创业扶持政策，提升就业创业技能培训实效，重点解决好高校毕业生、零就业家庭、退役军人、农民工等群体就业，做好农民工工资治欠保支工作。全面推行“五险合一”和机关事业单位养老保险统一经办管理，提高企事业单位退休人员基本养老金，建档立卡贫困户住院报销比例达到85%以上。深入实施全民参保计划，确保养老保险法定人群全覆盖。健全社会救助制度，做好留守儿童、空巢老人、残疾人等弱势群体关爱服务工作，重视发展农村养老事业，改造提升一批城乡养老机构。完善住房市场体系和住房保障体系，着力解决城镇低收入群体住房困难。

创新社会治理体系。完善基层群众自治制度，支持肃州区创建“全国社区治理和服务创新试验区”。坚持和发展新时代“枫桥经验”，健全社会矛盾源头预防、排查预警、多元化解机制，推动信访诉求法治化。加大安全生产、交通安全隐患排查整治，保障人民群众生命财产安全。加强产品质量、食品药品和农产品质量安全监管，积极创建省级食品安全城市和农产品质量安全县。深入开展“七五”普法，加快社会诚信体系建设。做好民族地区对口帮扶，推进全国民族团结进步示范市创建工作。强化社会面整体防控，持续开展扫黑除恶专项斗争，严厉打击黄赌毒、电信诈骗、涉众型经济犯罪等违法活动，加快“雪亮工程”建设，健全社会治安防控体系，确保社会大局和谐稳定。

各位代表！

利民之事，丝发必兴。市政府将围绕群众更加关心、更加直接的诉求期盼，办好十件为民实事：组织1000名高校毕业生进企业见习就业；建成酒泉第八中学和附设幼儿园，新增招生学位2460个；新建市中医院住院部综合楼，新增医疗床位200张；改造棚户区住房2.3万户；新建改造农村道路500公里；提升规范农民专业合作社，创建省级示范社50家、市级示范社150家；为2.64万名农村妇女进行“两癌”免费检查；新建改建城乡公厕150座、农村户用卫生厕所1.9万户；开展城乡文化惠民演出500场次以上；新增城区公共停车位2000个。

加强政府建设

新时代、新使命对政府自身建设提出新要求。我们要旗帜鲜明讲政治，驰而不息转作风，持之以恒提效能，做到为民、务实、清廉，努力打造人民满意政府。

以更高站位加强政治建设。高举习近平新时代中国特色社会主义思想伟大旗帜，树牢“四个意识”，坚定“四个自信”，做到“两个维护”，认真开展“不忘初心、牢记使命”主题教育。坚决贯彻中央大政方针，认真落实省委、省政府和市委各项决策部署，确保政令畅通、令行禁止。进一步肃清王三运、马光明流毒和影响，持续净化政治生态。严守政治纪律和政治规矩，自觉接受党委领导，主动请示汇报工作，确保政府工作保持正确政治方向。

以更强本领提升履职能力。按照增强“八个本领”、锤炼“五个过硬”的要求，深入学习研究新政策、新业务，提高执行政策、用好政策的能力水平，切实把上级的政策措施转化为推动酒泉发展的新实践、新动能、新支撑。着力破除思维定势和工作惯性，敢于开拓创新，善于干事成事，对上积极争取、对外合作招商、对内狠抓落实，推动全市工作争先进位。

以更大魄力强化责任担当。大力弘扬新时代铁人精神，激励广大干部勇于担当作为，增强斗争本领，直面问题，动真碰硬，在重大项目建设、现代产业培育、乡村振兴战略实施、生态环保问题整改、改革深化推进、保障改善民生等方面，多出能成事的实招，少找干不成的借口，真抓实干、攻坚克难，以实际行动和工作业绩推动“八个走前列”。

以更优作风提高政府效能。加强和改进调查研究，大力倡导求真务实作风，增强解决问题的针对性和实效性，千方百计为基层、企业和群众排忧解难。持续整治“四风”问题新表现，坚决破除形式主义、官僚主义，杜绝“表态多调门高、行动少落实差”等现象，切实纠正“高接低放”“有去无回”“应付式落实”等问题，严肃问责庸懒散慢、不在状态、不作为慢作为等人和事，增强政府工作的执行力和落实力。

以更实举措推进依法行政。加强法治政府建设，自觉运用法治思维和法治方式深化改革、推动发展、化解矛盾，严格按照法定权限和程序行使权力，把政府工作全面纳入法治轨道。自觉接受市人大及其常委会监督、政协民主监督、社会舆论监督，认真办好人大代表建议和政协委员提案。推进城市管理、社会建设、公共服务等领域政府立法工作，完善决策机制，规范决策程序，提升决策科学化、民主化、法治化水平。

以更严要求坚持廉洁从政。认真履行全面从严治党主体责任和“一岗双责”，严格落实中央八项规定实施细则精神和省委、市委实施办法，深入推进中央、省委巡视反馈意见整改落实。强化审计监督，加强财政预算、工程建设、政府采购等重点领域和关键环节风险防控，加大脱贫攻坚、民生保障、涉农政策落实跟踪问效。自觉接受纪检监察监督，坚决纠正损害群众利益的不正之风和违纪违规行为，不断把政府系统党风廉政建设和反腐败工作推向深入。

各位代表！

新时代是奋斗的时代，走前列必须苦干实干加油干。让我们更加紧密团结在以习近平同志为核心的党中央周围，在省委、省政府和市委的坚强领导下，凝聚全市干部群众的智慧力量，锐意进取，真抓实干，奋发有为，为酒泉决胜全面小康社会、建设省域副中心城市、实现高质量发展作出新的更大贡献！
